# Metabolic engineering of *Corynebacterium glutamicum* for enhanced 5-aminolevulinic acid production via precise porphobilinogen synthase activity modulation

**DOI:** 10.1128/aem.02447-25

**Published:** 2026-02-09

**Authors:** Hongyan Zhang, Caizhi Wei, Fanglan Ge, Jiao Li, Wei Li, XinLan Huang, Yao Ren

**Affiliations:** 1 College of life Sciences, Sichuan Normal University 66331, Chengdu, People's Republic of China; Chalmers tekniska hogskola AB, Gothenburg, Sweden

**Keywords:** 5-aminolevulinic acid, *Corynebacterium glutamicum*, PBGS mutants, enzyme activity modulation and metabolic effects, metabolic engineering, high-yield strain construction

## Abstract

5-Aminolevulinic acid (5-ALA) is a valuable precursor for pharmaceuticals and agriculture, but its microbial production is limited by tight coupling to essential heme biosynthesis. Here, we introduce a systematic, activity-graded tuning strategy for porphobilinogen synthase (PBGS, encoded by *hemB*) to decouple 5-ALA synthesis from heme metabolism in *Corynebacterium glutamicum*. Guided by structural and functional analyses, PBGS variants with progressively reduced activities were constructed to investigate the quantitative relationship between enzyme activity, cell growth, and 5-ALA accumulation. Controlled attenuation of PBGS activity maintained essential metabolism while markedly enhancing 5-ALA accumulation and minimizing porphyrin by-products. The engineered strain FA3 [*hemB*(D128E), *hemA* overexpression] achieved an optimal balance of growth and productivity. Metabolomic profiling confirmed that PBGS downregulation primarily suppressed porphyrin biosynthesis with minimal impact on central carbon metabolism. Subsequent metabolic and process optimizations, including *gdhA* and *aceA* deletion, dynamic *rhtA* expression, and cultivation control, further boosted production to 14.44 g/L 5-ALA in shake-flask culture, representing the highest shake-flask titer reported to our knowledge for *C. glutamicum* under similar conditions. This work provides the first systematic dissection of PBGS activity-dependent metabolic regulation and demonstrates that graded control of an essential enzyme enables rational metabolic decoupling, offering a broadly applicable framework for robust, high-yield microbial production of valuable compounds.

**IMPORTANCE:**

5-Aminolevulinic acid (5-ALA) is an important precursor with pharmaceutical and agricultural applications, but microbial production is often constrained by its tight linkage to essential heme metabolism. Here, we systematically tuned porphobilinogen synthase activity to decouple 5-ALA accumulation from excessive porphyrin flux while maintaining cell growth. This strategy not only enabled the highest reported 5-ALA titer in *Corynebacterium glutamicum* but also highlights a broadly applicable framework for rationally engineering essential metabolic enzymes to achieve robust, high-yield microbial production of valuable compounds.

## INTRODUCTION

5-Aminolevulinic acid (5-ALA) is the universal precursor of porphyrin compounds, including heme and chlorophyll, and finds broad applications in medical, agricultural, and industrial fields (1, 2). In nature, 5-ALA is synthesized via the C4 and C5 pathways (3, 4), both of which are tightly linked to heme biosynthesis and subject to strict feedback regulation (5). Because of this coupling, the tight coupling of 5-ALA synthesis to essential heme metabolism presents a challenge for its microbial overproduction. In recent years, significant progress has been made in constructing high-yield 5-ALA-producing strains through metabolic engineering, dynamic regulation, and high-throughput screening (6). Model hosts such as *Escherichia coli* and *Corynebacterium glutamicum* have been extensively engineered using strategies, including screening of efficient ALAS variants, pathway optimization, precursor supply enhancement, and improvement of strain tolerance. To further overcome production bottlenecks, researchers have integrated systems metabolic engineering with evolutionary approaches, such as constructing mutant libraries combined with high-throughput screening (HTS) (7). For example, Wang et al. established an HTS platform in *E. coli* based on reactive oxygen species (ROS) signaling and cAMP level changes induced by 5-ALA, thereby obtaining a mutant strain with a record titer of 58.54 g/L (8). Zhou et al. further optimized four key metabolic targets using a genome-scale model of *E. coli*, ultimately achieving 63.4 g/L 5-ALA (9). Despite these advances, compared with the industrial production of amino acids, such as glutamate and lysine, 5-ALA biosynthesis still lags behind in productivity and yield, highlighting the need for strategies that can overcome intrinsic metabolic constraints.

Porphobilinogen synthase (PBGS, encoded by *hemB*) is a highly conserved metalloenzyme that catalyzes the condensation of two 5-ALA molecules into porphobilinogen, a key precursor in tetrapyrrole biosynthesis (10). PBGS is widely recognized as a major rate-limiting enzyme in 5-ALA overproduction. Downregulation of PBGS activity is a common approach to reduce precursor consumption and enhance 5-ALA accumulation. Strategies such as antisense RNA (11, 12), CRISPRi-mediated interference (13, 14), promoter replacement (15), and the introduction of ASV degradation tags have been employed (9). However, these methods often face constraints, including partial or uncontrolled suppression, off-target effects, or a lack of quantitative understanding of how different degrees of PBGS downregulation affect enzyme function and cell physiology. Previous studies in *E. coli* showed that severe PBGS impairment leads to growth arrest (16), whereas mutants retaining low residual activity (~10%) remain viable only with external 5-ALA supplementation (17), highlighting the intrinsic trade-off between reducing PBGS activity and maintaining cellular viability.

**Fig 1:**
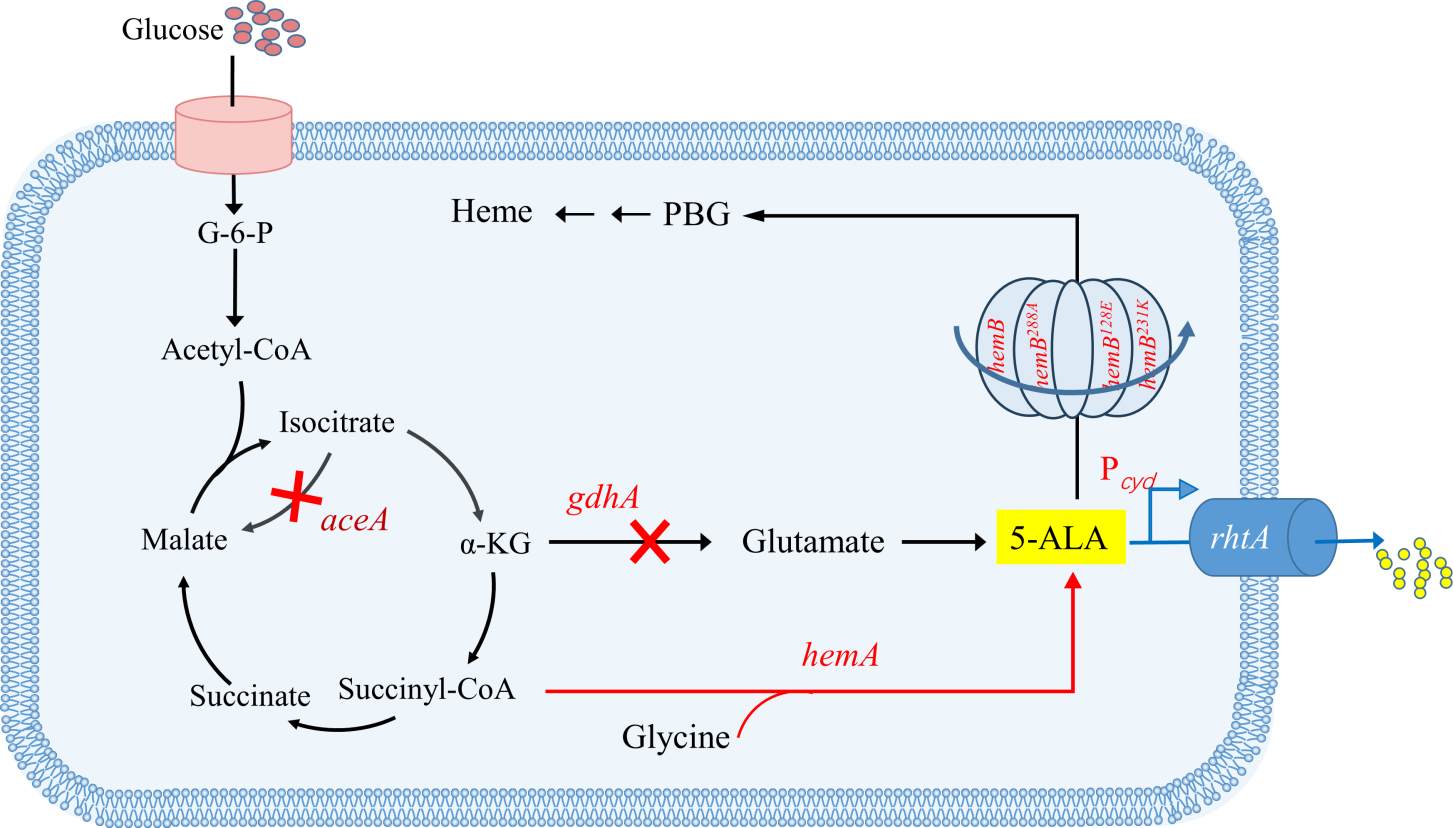
Metabolic engineering strategy for enhancing 5-ALA production in *C. glutamicum* F343. *aceA*, isocitrate lyase gene; *gdhA*, glutamate dehydrogenase gene; 5-ALA, 5-aminolevulinic acid; *hemB*, porphobilinogen synthase gene; PBG, porphobilinogen; *hemA*, 5-aminolevulinic acid synthase gene; *rhtA*, threonine/homoserine and 5-ALA efflux gene from *E. coli*.

In this study, building upon our structural and functional analysis of *C. glutamicum* PBGS, we constructed a panel of mutants spanning three distinct activity levels (medium, low, and extremely low). Their effects on cell physiology, biochemical characteristics, and metabolic networks were systematically evaluated, enabling the identification of an optimal variant that combined robust growth with high 5-ALA productivity. To further optimize carbon flux, we sequentially deleted *gdhA* (glutamate dehydrogenase, to reduce diversion toward non-target metabolites) and *aceA* (isocitrate lyase, to minimize competition between the glyoxylate shunt and the tricarboxylic acid [TCA] cycle). Complementing these strategies, a promoter with dynamic regulatory capacity was selected to drive efflux transporter expression, thereby further enhancing 5-ALA accumulation. Under shake-flask cultivation, the engineered strain achieved a 5-ALA titer of 14.44 g/L, setting a new record for *C. glutamicum* under similar conditions. To our knowledge, this is the first study to systematically analyze PBGS activity variants and apply them to build high-ALA-producing strains. Together, these results highlight a rational strategy to moderately reduce heme biosynthesis while efficiently increasing pyrrole intermediate accumulation (Fig. 1).

## MATERIALS AND METHODS

### Strains and plasmid construction

All strains, plasmids, and primers involved in this study are listed in Tables 1 and 2; Table S1. pK18Δ*gdhA* and pK18Δ*aceA* were obtained, respectively, by inserting upstream and downstream fragments of the glutamate dehydrogenase gene *gdhA* and the isocitrate lyase gene *aceA* from *C. glutamicum* into pK18mobSacB, for the purpose of deleting those genes (18). The expression plasmid pXMJ19*hemA* carries the *hemA* gene from *Rhodobacter capsulatus* ATCC 11166 (12).

**TABLE 1:** Strains used in this study

Strains	Description	Sources
*E. coli* BL21(DE3)	Competent wild-type strain	Beijing Transgen Biotech Co., Ltd.
*E. coli* BL21(DE3)-pET28a(+)-*cghemB*	Heterologous expression host	Laboratory stock
*E. coli* BL21(DE3)-pET28a(+)*-cghemB*-S288A	Heterologous expression host	Laboratory stock
*E. coli* BL21(DE3)-pET28a(+)*-cghemB*-D128E	Heterologous expression host	Laboratory stock
*E. coli* BL21(DE3)-pET28a(+)*-cghemB*-R231K	Heterologous expression host	Laboratory stock
Strain F1	Wild-type *C. glutamicum* F343	Laboratory stock
Strain F2	*C. glutamicum* F343 A288	This work
Strain F3	*C. glutamicum* F343 E128	This work
Strain F4	*C. glutamicum* F343 K231	This work
Strain F5	*C. glutamicum* F343 E128 *∆gdhA*	This work
Strain F6	*C. glutamicum* F343 E128 *∆aceA*	This study
Strain F7	*C. glutamicum* F343 E128 *∆gdhA ∆aceA*	This work
Strain F8	*C. glutamicum* F343 E128 *∆gdhA ∆aceA* P*_cyd_-rhtA*	This work
Strain F9	*C. glutamicum* F343 P*_cyd_-sfGFP*	This work
Strain FA1	*C. glutamicum* F343 *hemA*	This work
Strain FA2	*C. glutamicum* F343 *hemA* A288	This work
Strain FA3	*C. glutamicum* F343 *hemA* E128	This work
Strain FA4	*C. glutamicum* F343 *hemA* K231	This work
Strain FA5	*C. glutamicum* F343 *hemA* E128 *∆gdhA*	This work
Strain FA6	*C. glutamicum* F343 *hemA* E128 *∆aceA*	This work
Strain FA7	*C. glutamicum* F343 *hemA* E128 ∆*gdhA* ∆*aceA*	This work
Strain FA8	*C. glutamicum* F343 *hemA* E128 ∆*gdhA* ∆*aceA* P_*cyd*_ -*rhtA*	This work

**TABLE 2:** Plasmids used in this study

Plasmids	Description	Sources
pET28a(+)	*E. coli* expressing vector, Kmr	Laboratory stock
pET28a(+)-*cghemB*	pET28a(+) harboring the *cghemB* gene	Laboratory stock
pET28a(+)-*cghemB-*S288A	pET28a(+) harboring the *cghemB* gene S288A	Laboratory stock
pET28a(+)-*cghemB-*D128E	pET28a(+) harboring the *cghemB* gene D128E	Laboratory stock
pET28a(+)-*cghemB-*R231K	pET28a(+) harboring the *cghemB* gene R231K	Laboratory stock
pXMJ19	Expression plasmid; P_tac_ promoter	Laboratory stock
pK18mobsacB	Integration plasmid of *C. glutamicum*	Laboratory stock
pXMJ19-*sfGFP*	Removed lacIq from pXMJ19, constitutively expressing *sfGFP*, *CmR*	Laboratory stock
pXMJ19-*hemA*	pXMJ19 harboring the gene *hemA*	Laboratory stock
pXMJ19-P*_cyd_-sfGFP*	pXMJ19 harboring the gene P*_cyd_-sfGFP*	Laboratory stock
pK18-S288A	pK18 harboring the *cghemB* gene A288	This work
pK18-D128E	pK18 harboring the *cghemB* gene E128	This work
pK18-R231K	pK18 harboring the *cghemB* gene K231	This work
pK18-*∆gdhA*	Knock out the gene of *gdhA*	Laboratory stock
pK18-*∆aceA*	Knock out the gene of *aceA*	Laboratory stock
pK18-P*_cyd_-rhtA*	pK18 harboring the gene P*_cyd_-rhtA*	This work

A *hemB* gene fragment carrying a mutation at position 288 (19), synthesized by BGI (Beijing, China), was double-digested with *Bam*HI and *Hin*dIII, then ligated into the same enzymes-digested vector pK18mobsacB to construct the plasmid pK18*hemB* S288A. Plasmids pK18*hemB* D128E and pK18*hemB* R231K were constructed in the same way. Separately, a P*_cyd_rhtA* fragment synthesized by BGI was digested with *Eco*RI and *Hin*dIII and inserted into pK18mobsacB, yielding pK18P*_cyd_rhtA*. A P*_cyd_sfGFP* fragment was synthesized (20), digested with *Eco*RV and *Hin*dIII, and inserted into pXMJ19*sfGFP*, yielding pXMJ19P*_cyd_sfGFP*.

*C. glutamicum* F343 was transformed by electroporation, following the procedure described by Tauch et al. (21). After two rounds of screening, a strain carrying the mutation at position 288 was obtained according to the method of Schäfer et al. (22). Transformants were picked for PCR verification and sent for sequencing to confirm that the mutation at position 288 was successful. The other strains, F3–F8, were constructed in a similar manner. The plasmid pXMJ19*hemA* was electroporated into F1–F8, yielding recombinant strains FA1–FA8.

### Growth conditions and shake flask cultivation

*E. coli* DH5α was cultivated aerobically in LB broth (10 g/L tryptone, 5 g/L yeast extract, 10 g/L NaCl) at 37°C. For *C. glutamicum* cultivation and transformation, BHI medium (5 g/L peptone, 2.5 g/L yeast extract, 5 g/L NaCl, 18.5 g/L brain–heart infusion, 91 g/L D-sorbitol) was used.

For 5-ALA production, single colonies were inoculated into seed medium (LB supplemented with 20 g/L glucose and 3 g/L corn steep liquor) and grown overnight at 30°C, 220 rpm. A 1.5 mL inoculum was transferred into 50 mL CGXII medium [25 g/L glucose, 1 g/L KH_2_PO_4_, 1 g/L K_2_HPO_4_, 21 g/L MOPS, 10 g/L (NH_4_)_2_SO_4_, 7.5 g/L yeast extract, 0.25 g/L MgSO_4_, 0.01 g/L CaCl_2_, 0.01 g/L MnSO_4_, 1 mg/L ZnSO_4_·7H_2_O, 0.2 mg/L CuSO_4_, 0.4 mg/L biotin], and cultivated at 30°C, 220 rpm. After 4 h, 0.1 mM IPTG was added to induce gene expression, along with 7.5 g/L glycine. During fermentation, the pH was maintained at 6.4–7.0 using 25% ammonia water.

For cell growth and glucose consumption analysis, single colonies were cultured overnight in BHI and then inoculated at 3% (vol*/*vol) into modified CGXII medium (supplemented with 6.8 mM L-glutamate). Samples were collected every 4 h. Antibiotics were added when necessary (kanamycin 50 μg/mL, chloramphenicol 10 μg/mL).

### Molecular docking

Homology templates were searched in the SWISSMODEL database, and multiple sequence alignment and homology modeling were performed using Discovery Studio. The 3D structure of the ligand 5ALA was taken from the PubChem database and preprocessed with MGTools 1.5.6, including removal of water molecules, addition of hydrogen atoms, charge calculation, and merging of nonpolar hydrogens. Docking of the receptor and ligand was carried out with AutoDock Vina 1.2.3; the binding site was determined from the cocrystallized ligand of the homology template. The best conformation was visualized and analyzed using PYMOL.

### Molecular dynamics simulation

A 100 ns molecular dynamics (MD) simulation was carried out using the GROMACS 2020.6 software package to verify the reliability of the docking results. The protein topology was generated with the CHARMM36m force field (23). The protein was placed at the center of a cubic box with the box edge at least 1.0 nm from the protein, and Na^+^ and Cl⁻ ions were added to neutralize the net charge of the system. Energy minimization was performed using the steepest descent method, followed by a 100 ns NPT ensemble MD simulation. Trajectory analyses included root-mean-square deviation (RMSD), root-mean-square fluctuation (RMSF), radius of gyration (*R*_g_), solvent-accessible surface area (SASA), and hydrogen-bond analysis.

### Enzyme activity and kinetic parameter determination

Expression, purification, and activity assays of the *C. glutamicum hemB* gene (*cghemB*) and its mutants were carried out as reported by Zhu et al. (19). Kinetic parameters were measured under the following conditions: a 250 μL reaction containing 50 mM TrisHCl (pH 8.0), different concentrations of 5-ALA (0.0625–1 mM), and 10 μg of purified enzyme. Definition of enzyme activity unit: the amount of enzyme needed to produce 1 µ mol porphobilinogen per hour was defined as a unit of enzyme activity (1 U) at 37°C (24). All kinetic data were obtained by monitoring the initial linear rate of product formation and fitted by nonlinear regression using GraphPad Prism 10.0 to calculate *K_m_* and *V_max_*.

### Stability assessment during serial passage

To evaluate genetic stability, strains FA1 and FA3 were serially passaged in CGXII medium at 30°C for a total of 10 generations. At each generation, during mid-log phase, 1 % (vol/vol) of the culture was transferred into fresh medium. Biomass samples were collected from generation 1 (initial) and generation 10, and stability was assessed by comparing their biomass (OD₆₀₀) and 5ALA production. If the differences between generation 10 and generation 1 in these indicators were less than 5 % , the strain was judged to have a stable phenotype during serial passage.

### Characterization of the P*_cyd_* promoter

The plasmid carrying the *sfGFP* reporter under the control of the P*_cyd_* promoter was introduced into *C. glutamicum* F343. Positive colonies were inoculated into 24-deep-well plates (1 mL BHI broth per well) and incubated overnight at 30°C and 220 rpm as seed cultures. Seed cultures were then inoculated (2% vol*/*vol) into CGXII medium supplemented with different concentrations of heme (0.05–20 mg/L) and cultivated at 30°C and 220 rpm for 15 h. Green fluorescence intensity was measured using a fluorescence spectrophotometer.

### Metabolomics analysis

Strains F343-*hemA* (control, CK) and the engineered strain (T) were first grown overnight in seed medium and then inoculated (3% vol/vol) into fermentation medium. Cultures were incubated at 32°C and 220 rpm for 40 h. The fermentation broth was freeze-dried and stored in liquid nitrogen. Metabolites were extracted with 1 mL methanol solution (methanol:water = 4:1, vol/vol). Samples were ground at −10°C and 50 Hz for 6 min using a cryogenic grinder, followed by extraction under low-temperature ultrasonication (5°C, 40 kHz) for 30 min. Supernatants obtained after centrifugation were used for analysis.

Metabolite profiling was performed on a Thermo Fisher UHPLC-Q Exactive HF-X system. Chromatographic conditions: HSS T3 column, injection volume 3 μL; mobile phase A: 95% water + 5% acetonitrile (0.1% formic acid), mobile phase B: 47.5% acetonitrile + 47.5% isopropanol + 5% water (0.1% formic acid); flow rate 0.40 mL/min; column temperature 40°C. Mass spectrometry conditions: positive/negative ion mode, scan range 70–1,050 m/z; spray voltage +3,500 V/−3,500 V; sheath gas 50 arb, auxiliary gas 13 arb, source temperature 450°C; stepped collision energy 20–40–60 V.

### Data processing

Raw LC–MS data were processed with Progenesis QI for peak detection, correction, and alignment, and metabolites were identified using HMDB, Metlin, and an in-house database. Data matrices were uploaded to the Majorbio Cloud platform, filtered using the 80% rule for missing values, imputed, normalized by total ion current, and variables with QC RSD >30% were removed. Data were then log10-transformed.

### Statistical and pathway analysis

PCA was performed using the R package ropls. Significant differential metabolites were identified based on OPLS-DA VIP scores (>1) and Student’s *t*-test (*P* < 0.05). Differential metabolites were annotated against the KEGG database, and pathway enrichment was performed using Fisher’s exact test in Python (scipy.stats). Results were visualized with Venn diagrams (distribution of differential metabolites), heatmaps, and hierarchical clustering (metabolite expression patterns).

### Analytical methods

Cell biomass was measured at 600 nm using a UV spectrophotometer (Analytik Jena, Germany). Glucose and lactate concentrations were determined with an SBA-40C biosensor (Shandong Academy of Sciences, China).

5-ALA concentrations were quantified by the Ehrlich’s reagent method (25). Briefly, culture broth was centrifuged at 12,000 rpm for 3 min, and the supernatant was appropriately diluted. Diluted samples were mixed with sodium acetate buffer (pH 4.6) and acetylacetone, heated in boiling water for 15 min, cooled to room temperature, and reacted with modified Ehrlich’s reagent for 25 min. Absorbance was measured at 554 nm. PBG was measured similarly by mixing the diluted supernatant directly with Ehrlich’s reagent and reading absorbance at 554 nm after 25 min.

Heme concentration was determined fluorometrically (26). Diluted supernatants were mixed with 2 M oxalic acid, heated at 95°C–98°C for 30 min, cooled to room temperature, and fluorescence was measured at excitation 400 nm and emission 666 nm. Non-heated samples were used as controls, and fluorescence difference values were calculated.

### Statistical analysis

All experiments were performed in triplicate. Data are expressed as mean ± standard deviation (SD). Statistical analyses were conducted using GraphPad Prism 10.0 software.

## RESULTS AND DISCUSSION

### Enzymatic characterization and structural-dynamic analysis of PBGS mutants

Compared with wild-type (WT) PBGS, the enzymatic activities of the S288A and D128E mutants decreased to 15.5% and 5.17%, respectively, while R231K retained only trace activity (19). Kinetic analysis revealed *V_max_* values of 71.33, 13.07, and 4.262 μmol·h⁻¹·mg⁻¹ for WT, S288A, and D128E, respectively, with corresponding *K_m_* values of 0.252, 0.4225, and 0.3876 mM. These results indicate that all mutations substantially reduced catalytic efficiency, as evidenced by markedly decreased *V_max_* and moderately elevated *K_m_*.

**Fig 2:**
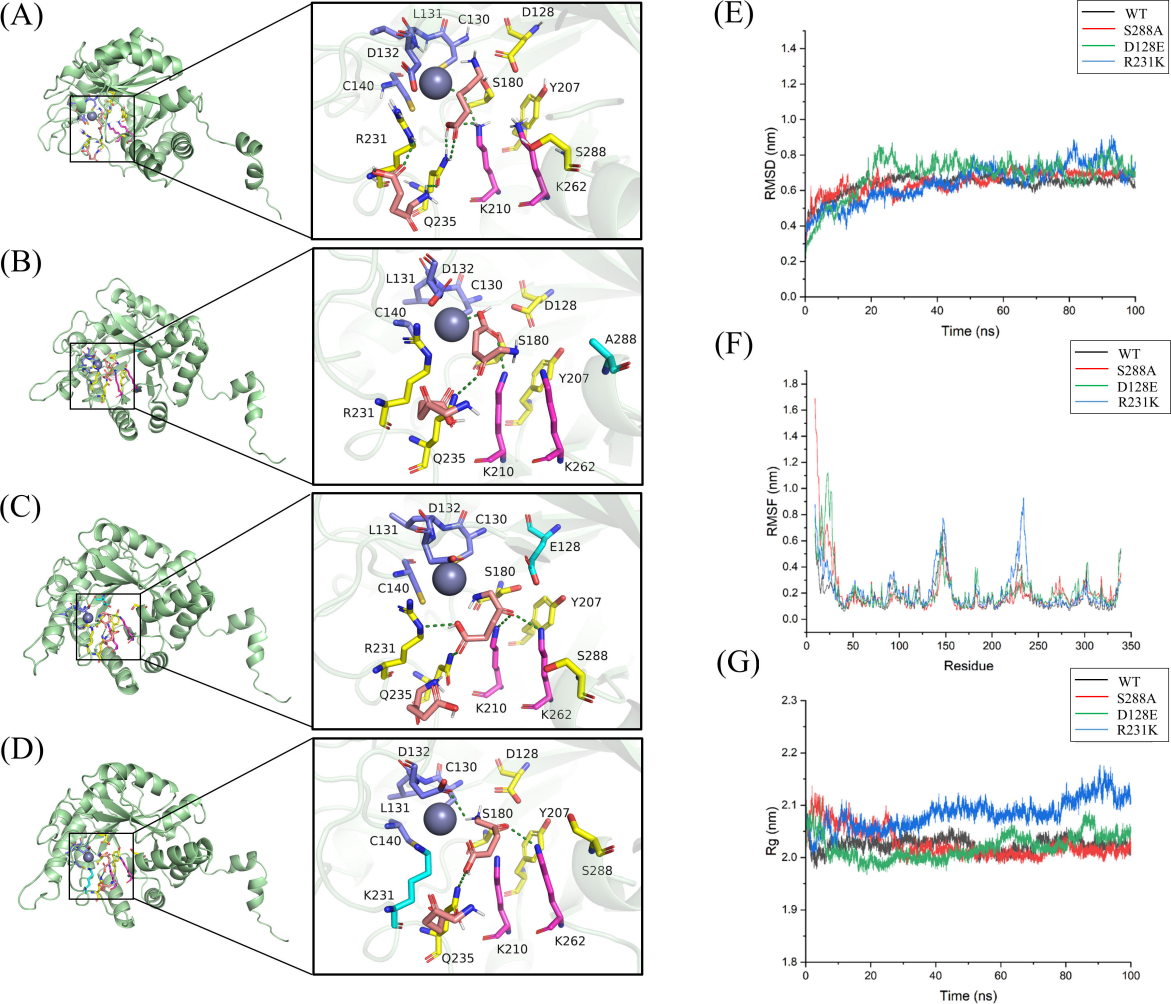
Effects of graded *hemB* downregulation on the physiological and biochemical properties of *C. glutamicum*. (**A**) Docking model of a single PBGS subunit with two substrate molecules. (**B–D**) Interactions between the mutated amino acid residues and surrounding residues, as well as the substrate. Gray spheres represent zinc ions. The two pink sticks indicate the substrate molecules, 5-ALA. Blue denotes the Zn²^+^ binding site, yellow indicates the active site, and the purple carbon backbone at these positions interacts with the Schiff base. (**E**) RMSD of the protein scaffold relative to the initial structure over the entire simulation. (**F**) Per-residue RMSF, reflecting local flexibility. (**G**) Time evolution of the radius of gyration (*R*_g_) as a measure of global compactness.

Molecular docking analysis elucidated the structural basis of the activity loss: in S288A, the absence of the hydroxyl group disrupted the (α/β)₈-barrel structure (Fig. 2B), S288A does not interfere with Zn²^+^ binding, as partial activity can be restored by Zn²^+^ addition; in D128E, the repositioned carboxyl group weakened key polar interactions (Fig. 2C), D128E likely affects the Zn²^+^ coordination microenvironment since its activity does not respond to Zn²^+^ supplementation; and in R231K, the missing imino group abolished critical polar contacts (Fig. 2D). Binding energy calculations further showed decreased substrate affinity, with binding energies of −7.632, −7.285, −7.408, and −6.864 kcal·mol⁻¹ for WT, S288A, D128E, and R231K, respectively.

Protein structural stability was assessed using RMSD, RMSF, and *R*_g_, where RMSD generally inversely correlates with stability (27, 28). As shown in Fig. 2E, all systems equilibrated after 50 ns, with the WT exhibiting the lowest mean RMSD (0.6543 ± 0.0186 nm), followed by progressively higher values for S288A, D128E, and R231K (0.6922 ± 0.0224, 0.7167 ± 0.0425, 0.7281 ± 0.0555 nm), indicating a stepwise decrease in rigidity. RMSF and *R*_g_ analyses (Fig. 2F and G) revealed that R231K exhibited markedly increased flexibility and conformational dynamics, whereas S288A and D128E showed only moderate local fluctuations. Similarly, R231K displayed the highest SASA and largest fluctuations (File S1), reflecting pronounced exposure of the hydrophobic core.

Hydrogen-bond analysis indicated that reduced bond numbers and increased fluctuations compromise enzyme-substrate stability. Among the mutants, D128E formed fewer and less stable hydrogen bonds, while R231K exhibited excessive fluctuations that disrupted the hydrogen-bonding network (Fig. S2).

Collectively, these analyses clarify the structure-function relationships of PBGS mutants. S288A, D128E, and R231K all perturb the catalytic center, with R231K causing the most severe structural distortion. While S288A and D128E exert relatively limited effects on overall conformation, D128E induces more pronounced local fluctuations and hydrogen-bond loss. These findings provide atomic-level insights into the mechanistic roles of key residues in PBGS catalysis and offer a theoretical basis for rational enzyme design and engineering.

### Construction of PBGS activity-reduced mutants and their physiological impacts

**Fig 3:**
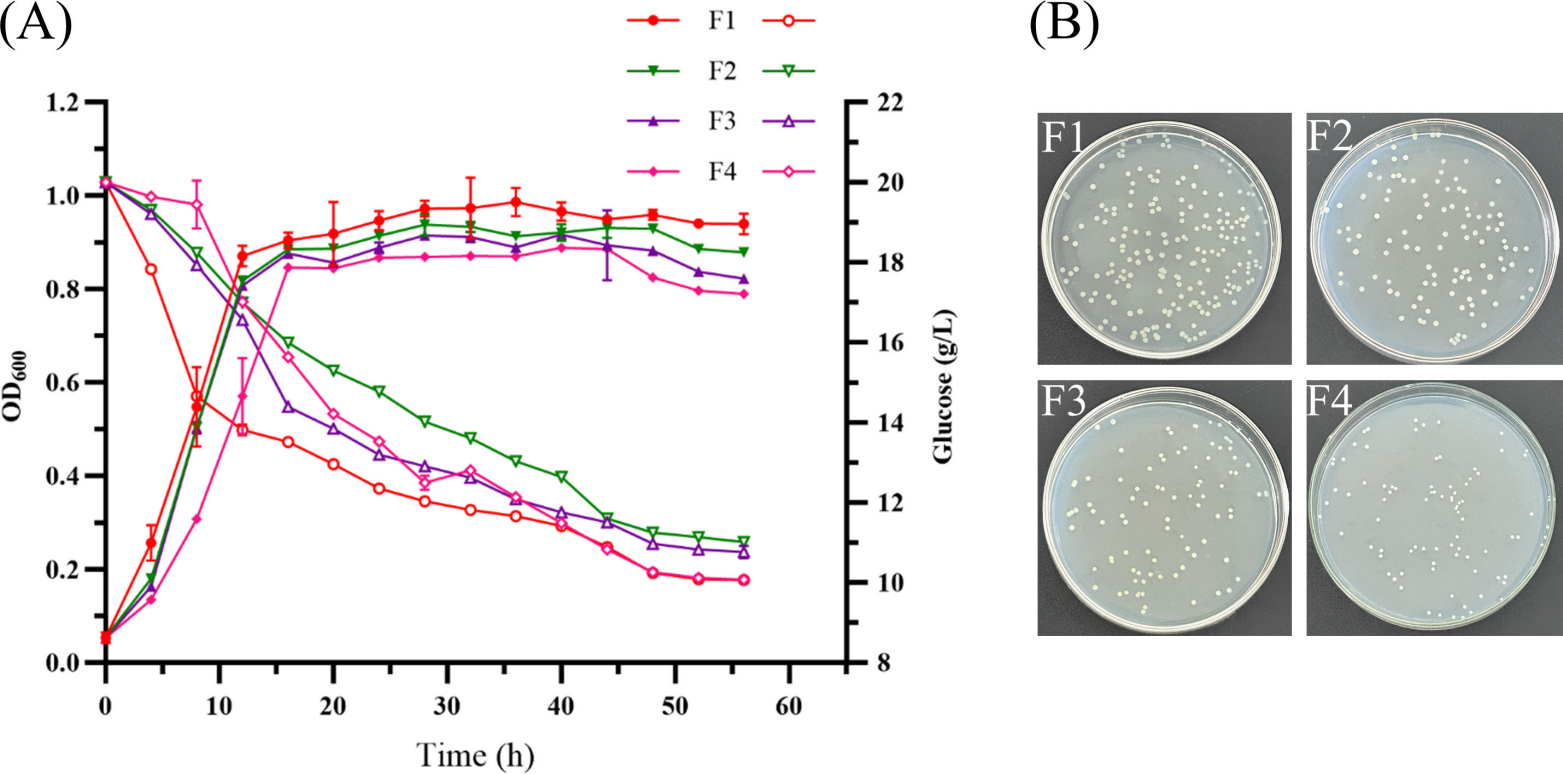
(**A**) Growth curves and glucose consumption of the wild-type and mutant strains. (**B**) Plate images of wild-type and mutant strains at the same dilution.

PBGS is the sole enzyme channeling 5-ALA into the heme biosynthetic pathway. Based on semi-rational engineering of three key mutations (A288, E128, and K231), we constructed the corresponding *C. glutamicum* mutant strains F2, F3, and F4 via gene replacement. Enzymatic assays showed that, although PBGS activity in the wild-type strain was relatively low, all mutant strains exhibited a stepwise decrease in activity, further confirming that these mutations impair the catalytic function of PBGS. Growth assays in CGXII medium revealed no significant lag phase for any strain (Fig. 3A). F1–F3 exhibited logarithmic growth between 1 and 12 h, whereas F4 extended to 16 h. Final OD₆₀₀ values were 0.98, 0.93, 0.91, and 0.88, respectively. Glucose consumption rates were slower in mutants early on, but all strains ultimately consumed ~10 g/L. Colony morphology showed that F2 and F3 resembled F1, while F4 colonies were notably smaller (1.46 mm vs 2.56 mm; Fig. 3B).

These results demonstrate that site-directed mutagenesis enables graded reduction of PBGS activity, with proportional effects on host growth and metabolism. A positive correlation was observed between PBGS activity and growth; however, even the >99% reduction in F4 still permitted near-normal growth, suggesting that minimal PBGS activity is sufficient to sustain essential heme biosynthesis. This contrasts with earlier findings where PBGS-null mutants exhibited severe defects even with exogenous 5-ALA supplementation (16). Compared with transcriptional regulation methods such as RNAi or CRISPRi (13, 14), direct enzyme mutagenesis provides stable, sequence-intrinsic control without requiring additional regulatory elements, offering a broadly applicable strategy.

### Impact of PBGS attenuation on 5-ALA accumulation and heme biosynthesis

**Fig 4:**
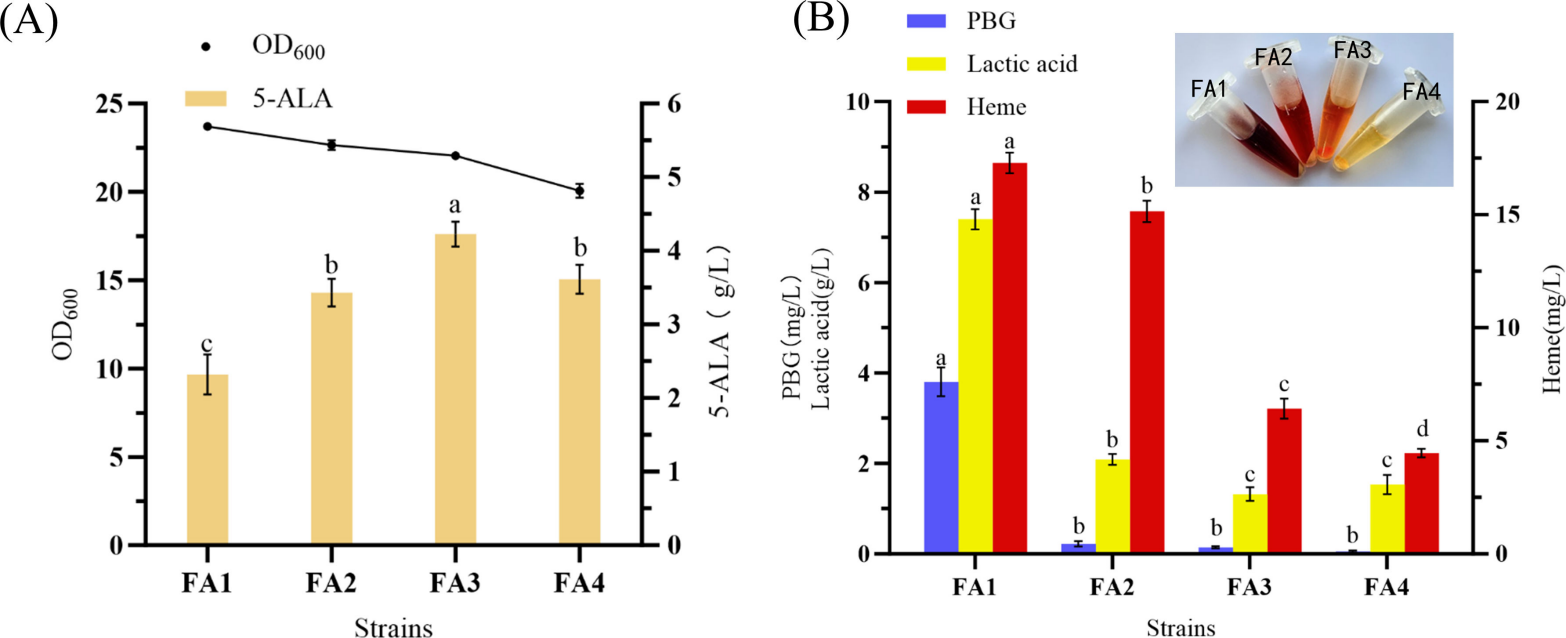
Effects of down-regulation of *hemB* on biomass, 5-ALA, and downstream products. (**A**) Biomass and 5-ALA production. (**B**) Comparison of porphobilinogen, heme, lactic acid content, and the color of fermentation broth of different strains. Different letters (a–d) above the bars indicate statistically significant differences among groups (one-way ANOVA, *P* < 0.05).

To evaluate the effect of PBGS weakening on 5-ALA production, a plasmid carrying *hemA* was introduced into strains F1–F4, yielding FA1–FA4. The parental wild-type strain F343 (strain F1) was also measured for 5-ALA and heme levels; both were negligible, confirming that 5-ALA production is largely dependent on *hemA* overexpression and PBGS modulation. After 48 h induction, 5-ALA titers reached 2.32, 3.43, 4.22, and 3.61 g/L, respectively (Fig. 4A). All PBGS mutants showed significantly increased 5-ALA accumulation compared to FA1, with FA3 achieving the highest improvement (1.82-fold). Biomass analysis indicated OD₆₀₀ values of 23.71, 22.66, 22.05, and 20.07, with only FA4 showing a marked reduction.

Fermentation broth coloration varied notably: FA1 appeared dark reddish-brown, while FA2 and FA3 progressively lightened, and FA4 displayed pale yellow (Fig. 4B). This correlated with decreased porphyrin accumulation due to reduced PBGS activity. Quantification of pathway intermediates confirmed that PBG levels in FA2–FA4 were reduced by 94.2%, 96.3%, and 98.7%, respectively, compared with FA1. Heme levels dropped from 17.29 mg/L (FA1) to 15.15, 6.42, and 4.46 mg/L in FA2–FA4, indicating substantial inhibition of downstream biosynthesis. Lactic acid byproduct accumulation was also markedly reduced, from 7.40 g/L (FA1) to 2.08, 1.32, and 1.53 g/L, likely reflecting enhanced respiratory flux and reduced overflow metabolism in mutants.

Previous studies improved 5-ALA yields by repressing *hemB* expression or promoting PBGS degradation, achieving modest improvements (0.86–3.68 g/L) (8, 9, 13). However, systematic quantitative evaluation of PBGS activity levels has been lacking. Here, site-directed mutagenesis effectively diverted carbon flux away from heme biosynthesis, lowered porphyrin accumulation, and enhanced 5-ALA production, while minimizing growth defects. Notably, FA3 achieved the highest 5-ALA production under conditions approaching the biomass growth level of the wild type, surpassing previously reported strains. In addition, FA3 showed strong genetic stability during continuous subculturing. Relative to the first generation, the tenth generation displayed only a 2.1% change in biomass and a 3.45% variation in 5-ALA production, both below 5%, indicating that its production traits remained stable throughout passaging. Unlike earlier PBGS mutants that retained only ~10% activity but suffered significant growth impairment (17), FA4 maintained limited growth and product formation despite its extremely low enzymatic activity, with a specific yield (0.18) only slightly lower than that of FA3 (0.19). This observation further highlights the remarkable metabolic robustness of *C. glutamicum*.

Collectively, these findings reveal a direct relationship between PBGS activity and host metabolism and demonstrate that precise mutational tuning enables fine control over essential enzymes. FA3, with an optimal balance between growth, substrate utilization, 5-ALA accumulation, and reduced byproduct formation, was selected as the chassis for further engineering. In this study, we for the first time reduced PBGS activity to the lowest level reported to date, effectively preventing excessive flux into heme and porphyrin by-products, while achieving the highest improvement in 5-ALA accumulation. This not only facilitates downstream purification but also provides new insights and references for the fine-tuning of heme biosynthesis and the efficient production of 5-ALA.

### Metabolomic insights into the impact of PBGS attenuation

**Fig 5:**
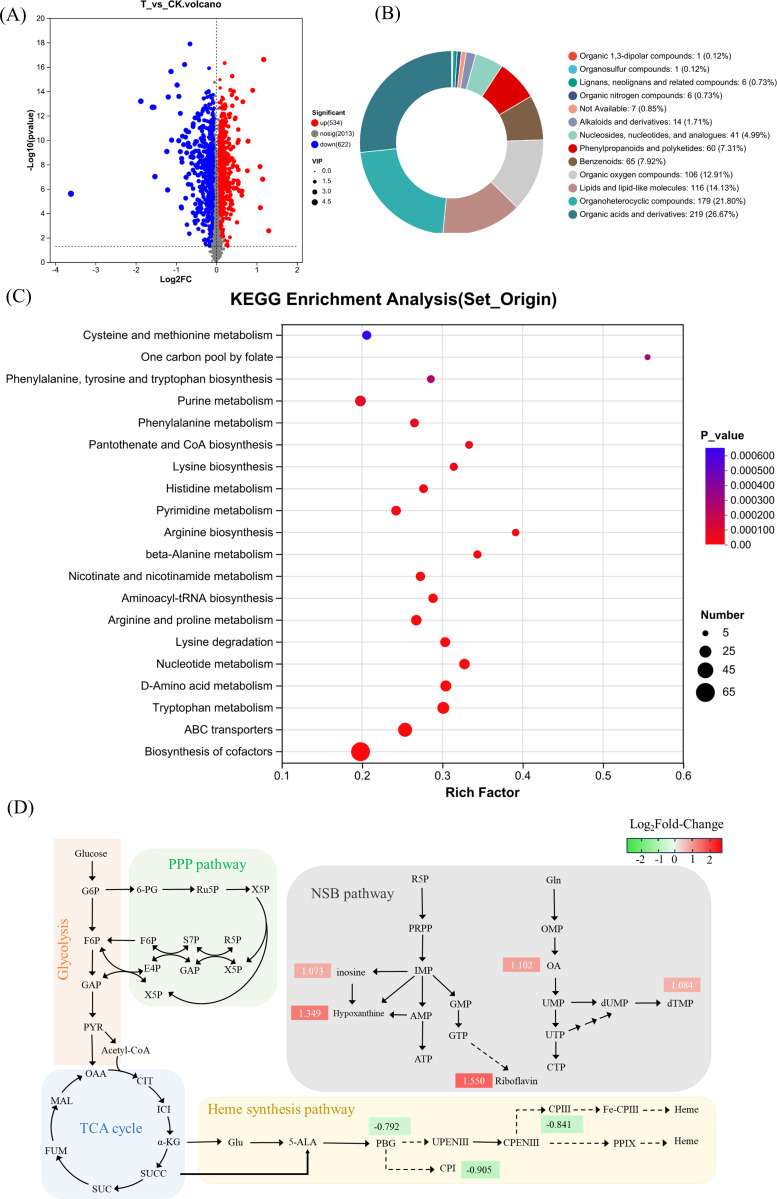
Metabonomics analysis. (**A**) Volcanic map of differential metabolite distribution. (**B**) Pie chart of differential metabolite classification. (**C**) KEGG enrichment pathway diagram. (**D**) Mapping of differential metabolites between mutant strain FA3 and control strain FA1. The color gradient depicts log₂ fold-change (FC): red, up-regulation (log₂FC > 0); green, down-regulation (log₂FC < 0). Metabolites with |log₂FC| ≥ 0.5 are regarded as significantly changed.

To investigate the metabolic consequences of reduced PBGS activity, metabolomic profiling was conducted on the control strain FA1 (CK group) and the PBGS mutant FA3 (T group). Importantly, both strains share the same *hemA* overexpression background, and the primary difference is the reduction of PBGS activity in FA3. A total of 1,156 differential metabolites were identified under the criteria VIP >1 and *P* <0.05, including 622 downregulated and 534 upregulated compounds (Fig. 5A). Although the differential metabolites spanned multiple chemical categories (Fig. 5B), pathway-level analysis offered a more biologically meaningful interpretation of the metabolic adaptations triggered by PBGS attenuation.

KEGG enrichment (Fig. 5C) highlighted several pathways with significant responses, notably cofactor biosynthesis, nucleotide metabolism, and specific amino acid-related processes. Mapping key metabolites to metabolic networks (Fig. 5D) showed that PBG, the direct product of PBGS, decreased by 0.792-fold, along with pronounced reductions in coproporphyrin I and III. Because coproporphyrin III is the oxidized form of coproporphyrinogen III—direct precursor of protoporphyrin IX and heme, these coordinated decreases demonstrated a clear bottleneck in the porphyrin pathway, supporting that PBGS attenuation, rather than *hemA* overexpression, predominantly drives these changes.

Beyond this primary effect, several secondary pathway-level adjustments suggested compensatory responses to partially reduced heme availability. Riboflavin levels increased 1.55-fold, consistent with enhanced FMN/FAD synthesis, a shift that may help stabilize electron transfer capacity when heme-dependent cytochromes are limited (29). Similarly, increased tetrahydrofolate and dihydrofolate indicated strengthened one-carbon metabolism, which supports redox balance and supplies precursors for nucleotide biosynthesis (30). Purine and pyrimidine pathways also showed elevated intermediates—including inosine, hypoxanthine, dTMP, and orotate—implying reinforced nucleotide production. Such upregulation may help sustain DNA replication, repair, and ATP-generating pathways under conditions where heme-based oxidative phosphorylation could be constrained. Although the enrichment analysis pointed to amino acid metabolism, the actual metabolite changes were small, suggesting a limited physiological impact.

Central carbon metabolism remained largely stable. Glycolysis, the pentose phosphate pathway, the TCA cycle, and most amino acid pathways showed minimal perturbation, indicating that PBGS attenuation does not broadly disrupt cellular energy or carbon flux. Instead, the cell appears to respond specifically to decreased porphyrin throughput by adjusting cofactor and nucleotide biosynthesis to preserve electron flow, biosynthetic capacity, and overall metabolic robustness. Despite upstream suppression, the metabolomics data set showed a relatively stable intracellular heme signal, suggesting that homeostatic mechanisms may buffer heme levels within a functional range. However, we acknowledge that metabolite pool changes alone cannot definitively establish flux alterations, and future studies integrating transcriptomics or isotope tracing are required to validate these pathway-level interpretations.

Therefore, under an identical *hemA* overexpression background, *hemB*(D128E) increased 5-ALA production primarily by limiting carbon flux into downstream porphyrin-consuming steps rather than by perturbing global metabolism. The observed adjustments in riboflavin, one-carbon, and nucleotide pathways likely represent targeted compensatory adaptations to maintain respiratory and biosynthetic functions under reduced PBGS activity. Collectively, these findings demonstrate that moderate PBGS downregulation can effectively enhance 5-ALA accumulation while preserving metabolic homeostasis, offering a mechanistic rationale for fine-tuning PBGS as a strategy for engineering high-yield 5-ALA producers.

### Blocking competing pathways to enhance 5-ALA synthesis

**Fig 6:**
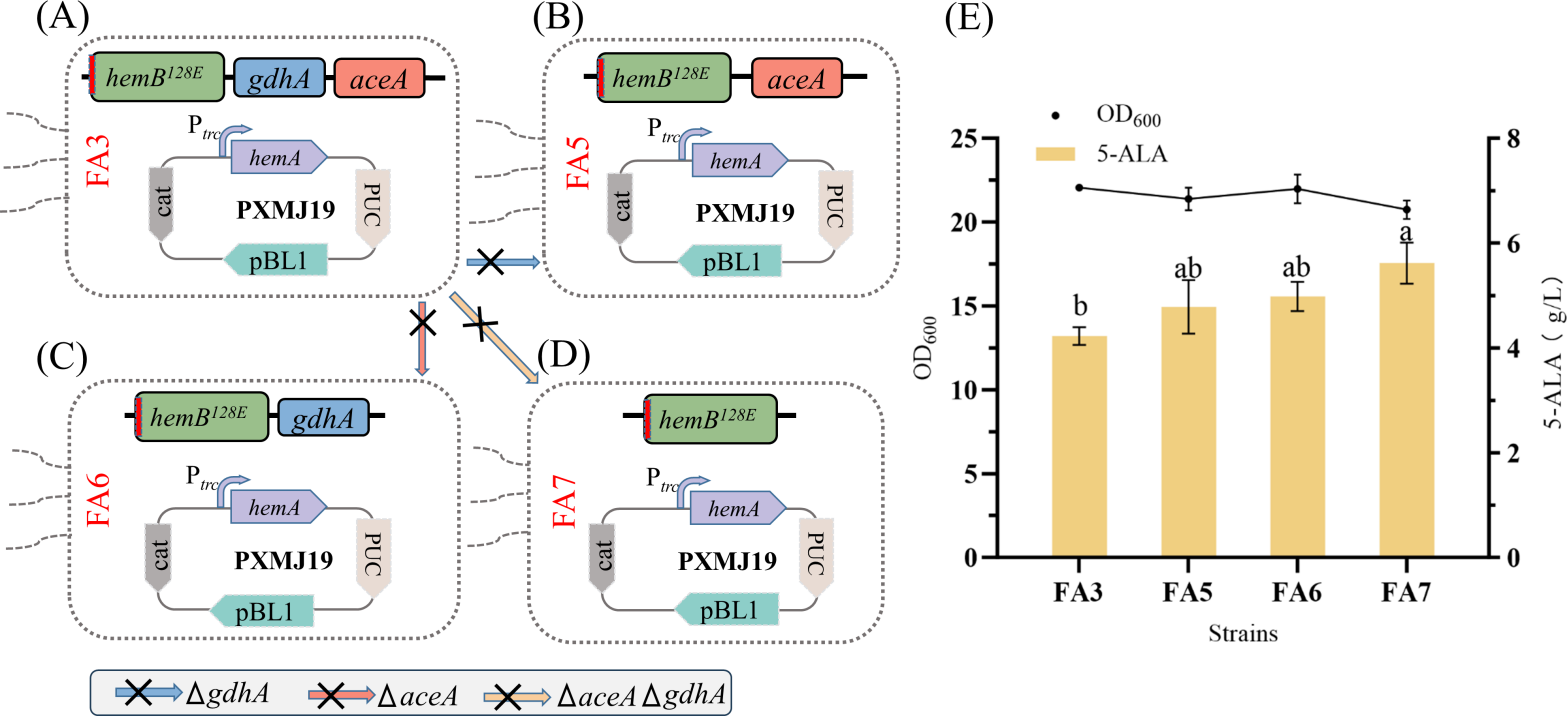
Enhancing 5-ALA synthesis by blocking upstream competitive branch pathways. (**A–D**) Schematic representation of the construction of *aceA* and *gdhA* knockout strains. (**E**) Effects of the knockouts on cell biomass and 5-ALA production. Different letters (a–b) above the bars indicate statistically significant differences among groups (one-way ANOVA, *P* < 0.05).

Succinyl-CoA is the key precursor of 5-ALA and is supplied primarily through the TCA and glyoxylate cycles (14). In the TCA cycle, α-ketoglutarate (α-KG) can be diverted to glutamate by glutamate dehydrogenase (GDH), while isocitrate can be cleaved by isocitrate lyase (*aceA*) into succinate and glyoxylate (31). To redirect carbon flux toward the TCA cycle and strengthen succinyl-CoA availability, *gdhA* and *aceA* were individually deleted, yielding strains FA5 and FA6 (Fig. 6A). Compared with FA3, FA5 and FA6 achieved 5-ALA titers of 4.67 g/L (1.10-fold increase) and 4.85 g/L (1.14-fold increase), without compromising growth (Fig. 6B). A double knockout (FA7) further boosted 5-ALA production to 5.36 g/L (1.27-fold increase). The 5-ALA production of FA5, FA6, and FA7 was all higher than that of FA3, with the difference between FA7 and FA3 reaching statistical significance (*P* < 0.05). These results demonstrate that coordinated disruption of *gdhA* and *aceA* effectively enhances succinyl-CoA supply and promotes 5-ALA biosynthesis.

Nevertheless, the high flux nature of the TCA cycle and the complexity of coenzyme A biosynthesis often render single-gene manipulations insufficient or destabilizing (5). Our previous studies revealed that *C. glutamicum* harbors two GDH isoenzymes, with *gdhA* exhibiting 1.62-fold higher affinity for α-KG than *gdhB* (32), and knockout studies confirmed that α-KG conversion is primarily mediated by *gdhA* (18). Thus, deleting *gdhA* significantly reduces α-KG consumption while retaining glutamate synthesis, avoiding growth defects. In line with this, the deletion of *gdhA* was found to reduce excessive glutamate formation and redirect carbon flux toward the TCA cycle, thereby increasing the availability of succinyl-CoA for 5-ALA biosynthesis. This observation is consistent with our previous metabolomic data showing upregulation of *icd* and *lpdA* and downregulation of *sucC* and *sucD*, collectively favoring succinyl-CoA accumulation (18). Meanwhile, *aceA* controls the branch point between the TCA and glyoxylate cycles; prior studies showed that *aceA* repression increased 5-ALA titers by 10.5%–15.0% (33). Consistent with these reports, *aceA* deletion redirected flux toward α-KG and succinyl-CoA, thereby boosting 5-ALA accumulation. The combined deletion of *gdhA* and *aceA* further reinforced this effect without impairing cell growth.

### Dynamic expression of efflux proteins

Enhancing the efflux of 5-ALA has been proven to be an effective strategy for increasing its production. The two-component system HrrSA senses intracellular heme levels, in which phosphorylated HrrA activates the transcription of the cytochrome bd complex α-subunit gene *cydA* (20). Based on this principle, we designed a heme-responsive dynamic regulatory system: as flux through the C4 pathway increases, heme concentration rises, thereby activating the P*_cyd_* promoter to drive the expression of the threonine/homoserine exporter *rhtA* and enable timely 5-ALA export.

**Fig 7:**
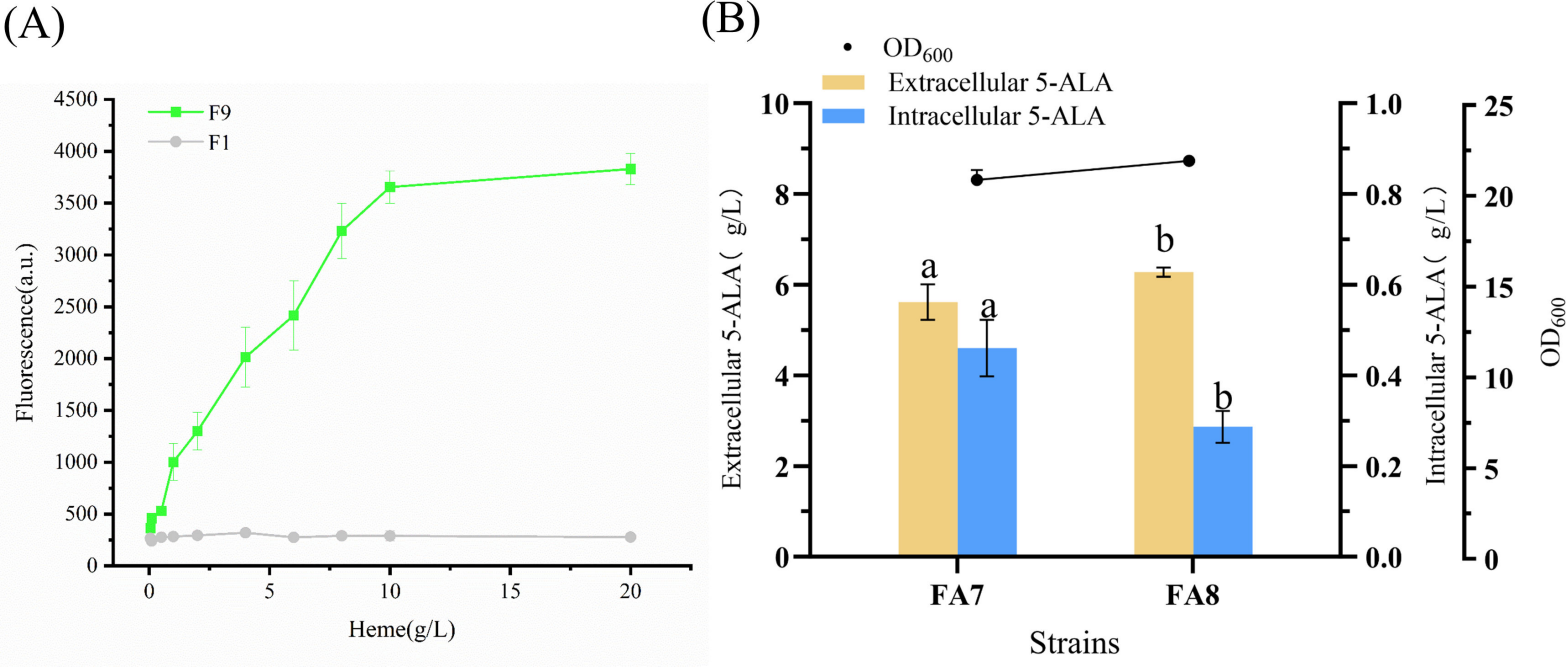
Dynamic regulation of efflux protein enhances 5-ALA synthesis. (**A**) Response of the P*_cyd_* promoter to varying heme concentrations. (**B**) Effects of efflux protein regulation on cell biomass and 5-ALA production. Different letters (a–b) above the bars indicate statistically significant differences among groups (one-way ANOVA, *P* < 0.05).

To evaluate the responsiveness of the P*_cyd_* promoter, green fluorescent protein (*sfGFP*) was used as a reporter. The native promoter of pXMJ19-*sfGFP* was replaced with P*_cyd_*, generating strain F9. Fluorescence assays under varying exogenous heme concentrations showed that F9 exhibited a strong increase in *sfGFP* intensity with rising heme levels, while the control strain F1 remained unchanged (Fig. 7A). Fluorescence enhancement was detectable at 1 mg/L heme and plateaued above 10 mg/L, confirming that P*_cyd_* responds sensitively to heme and achieves the intended dynamic regulation.

The P*_cyd_-rhtA* fragment was integrated into the FA7 genome via plasmid pK18-P*_cyd_-rhtA*, yielding strain FA8. Shake-flask fermentation (Fig. 7B) showed that, compared with FA7, intracellular 5-ALA in FA8 decreased from 0.46 to 0.28 g/L (−39%), while extracellular 5-ALA increased by 11.8% to 6.27 g/L, accompanied by higher cell biomass. These results indicate that the P*_cyd_*-driven *rhtA* expression system markedly enhances 5-ALA production efficiency.

Notably, excessive intracellular 5-ALA can induce ROS, impairing cell growth and membrane integrity (34). In *E. coli*, the exporter *rhtA* has been identified as a key 5-ALA efflux channel, preventing toxic accumulation (35). However, its broad substrate specificity may compromise selectivity (11, 36). To address this, Zhang et al. developed a heme-responsive auto-induction system for precise *rhtA* regulation, thereby facilitating 5-ALA export (37). Compared with static control, dynamic regulation offers greater potential for balancing metabolic fluxes and improving robustness under complex physiological conditions (38). In this study, despite its simplicity, the P*_cyd_*-based *rhtA* control system effectively promoted 5-ALA efflux in response to intracellular accumulation, alleviated feedback inhibition, and boosted production, providing a practical strategy for strain engineering.

### Optimization of fermentation conditions for 5-ALA biosynthesis

**Fig 8:**
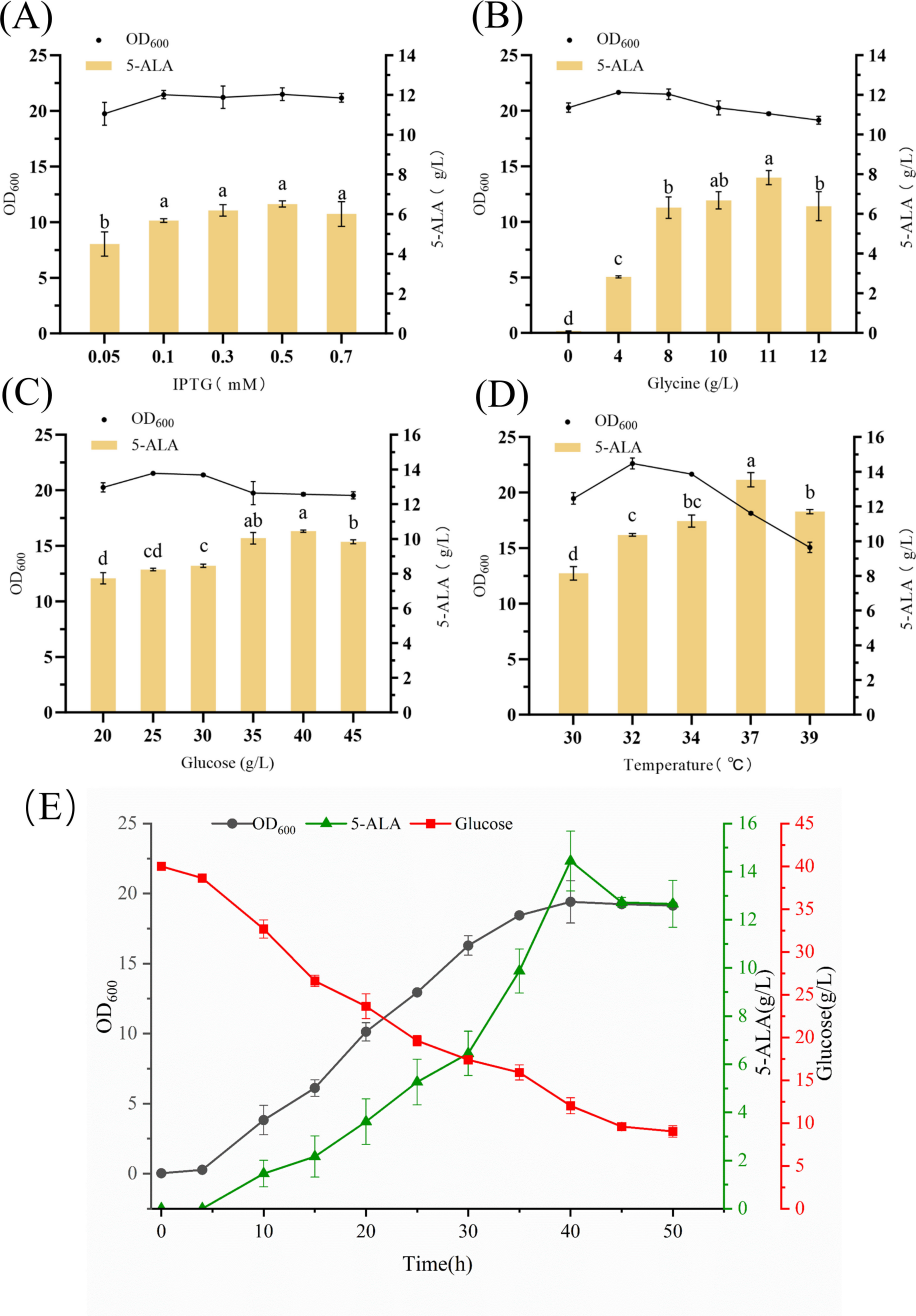
Effects of shake-flask fermentation optimization on the engineered strain FA8. (**A–D**) Influence of concentration of IPTG, glycine, glucose, and cultivation temperature on cell biomass and 5-ALA production. (**E**) Dynamic changes in cell biomass, glucose consumption, and 5-ALA content during fermentation under optimized conditions. Different letters (a–d) above the bars indicate statistically significant differences among groups (one-way ANOVA, *P* < 0.05).

To comprehensively assess the production potential of engineered strain FA8, medium components and fermentation parameters were optimized. IPTG concentration was first examined (Fig. 8A). Both insufficient and excessive IPTG suppressed 5-ALA accumulation, with the highest titer (6.51 g/L) obtained at 0.5 mM, which was therefore selected as the optimal induction level.

Glycine, the key substrate of the C4 pathway, strongly influenced both growth and production (Fig. 8B). At low concentrations, glycine positively correlated with 5-ALA accumulation, whereas concentrations above 8 g/L inhibited growth. Nevertheless, the maximum titer of 7.83 g/L was achieved at 11 g/L glycine, consistent with previous reports (35), suggesting that metabolic stress may restrict growth without impeding production.

As the primary carbon source, glucose concentration also markedly affected productivity (Fig. 8C). At 40 g/L glucose, 5-ALA reached its highest yield (10.44 g/L). However, cell growth was inhibited at concentrations above 30 g/L, likely due to by-product accumulation.

Temperature exerted dual effects on growth and product synthesis (Fig. 8D). At 37°C, 5-ALA titer peaked at 13.53 g/L, though biomass (OD_600_ = 19.13) was lower than at 32°C, where cells grew faster (OD_600_ = 22.61) but produced less 5-ALA. These results suggest that 32°C favors growth, while 37°C benefits product accumulation.

Accordingly, a two-stage temperature control strategy was developed: pre-induction (0–4 h) at 32°C to promote biomass accumulation, followed by 37°C to maximize production. Under the optimized conditions (0.5 mM IPTG, 11 g/L glycine, 40 g/L glucose), FA8 reached an OD_600_ of 19.24 and produced 14.44 g/L 5-ALA after 40 h, corresponding to a volumetric productivity of 0.36 g/L/h and a yield of 0.36 g/g glucose. Although these values were obtained under shake-flask conditions, they are already competitive compared with previously reported laboratory-scale systems. It is worth noting that industrial *E. coli* strains operating in fed-batch bioreactors can achieve titers exceeding 60 g/L, including the recent studies reporting 58–63 g/L through systematic metabolic engineering and high-throughput screening (9, 11). However, these high titers rely on controlled feeding, high-density cultivation, and optimized oxygen transfer that are not accessible in shake flasks. Given the robust performance of FA8 in simple batch cultures, the strain shows clear potential for further improvement and scale-up in fed-batch or bioreactor systems, where the dynamic PBGS regulation strategy could be readily integrated with controlled feeding and oxygen management to further enhance production (Fig. 8E).

Overall, the results demonstrate that glycine supplementation, C/N ratio adjustment, and phased temperature control are critical for balancing growth and production. The fermentation strategy not only improved 5-ALA yield but also enhanced its overall accumulation throughout cultivation, as reflected by the higher final titers achieved under the optimized conditions.

### Conclusions

This study established a quantitative framework for modulating PBGS (*hemB*) activity through multi-site mutations and demonstrated how graded enzyme attenuation can systematically reshape metabolic flux in *C. glutamicum*. Rather than complete gene disruption or transcriptional repression, precise tuning of an essential enzyme enabled balanced heme biosynthesis, minimized porphyrin overflow, and maximized 5-ALA accumulation. The key innovation of this work lies in integrating enzyme-level activity modulation with system-level flux balancing, providing a generalizable approach to control essential metabolic nodes. Coupled with rational precursor engineering and a dynamic efflux system, this strategy offers both mechanistic insight and practical feasibility for industrial 5-ALA fermentation. The same principle may be extended to optimize other bioprocesses where fine control of essential pathways is required.

## Data Availability

Metabolome raw data are openly available in Figshare at https://doi.org/10.6084/m9.figshare.31072981.

## SUPPLEMENTAL MATERIAL

Supplemental material aem.02447-25-s0001.docx Tables S1 and S2; Fig. S1 and S2.

ASM does not own the copyrights to Supplemental Material that may be linked to, or accessed through, an article. The authors have granted ASM a non-exclusive, world-wide license to publish the Supplemental Material files. Please contact the corresponding author directly for reuse.
