# Supplementary material for: Metabolic engineering of Corynebacterium glutamicum for enhanced 5-aminolevulinic acid production via precise porphobilinogen synthase activity modulation
Source: Appl Environ Microbiol. 2026 Feb 9;92(3):e02447-25. doi: 10.1128/aem.02447-25 (PMC12997798; doi:10.1128/aem.02447-25)
Supplement: Supplemental material — Tables S1 and S2; Fig. S1 and S2. [file aem.02447-25-s0001.docx]

Table S1 Primers used in this study

| Primers | Primers Nucleotidesequence(5 '-3 ') |
| --- | --- |
| Kan Check F | GATGGATTGCACGCAGGTTCTCCG |
| Kan Check R | GAGCGGCGATACCGTAAAGCACGAG |
| A288 Check F1 | CTCCCACGCACTAATTCGTCGTCCG |
| A288 Check R1 | CCTGAATCATCGCGTACTCACCTGC |
| A288 Check R2 | GCACCGGCACGCTTAATGGAGGTC |
| E128 Check F1 | CACTTCTTCTGATTACTCCCACGCAC |
| E128 Check R1 | GATCCGTGAACTCATCAAGGCAAGTT |
| E128 Check R2 | GAATGGTCCAAAGAAGGCTGATGCA |
| K231 Check F1 | GCACACGGAAGATTCACTGCTGCG |
| K231 Check R1 | GCAGGATCTTGCTGGTAGGTGCGCT |
| K231 Check R2 | AGCCACTGGGACAGGTGAGGTATC |
| *gdhA* check F | AGGCTGCGGCGATGTCAATGTAG |
| *gdhA* check R | CTTGAAGCCAGCAATGTTAGCGCC |
| *aceA* check F | TTTCTGGGATGGCGTTGGTGCCGTA |
| *aceA* check R | GTGGAACTGGCCTTCTTCAGTGGAA |
| P*_cyd_-rhtA* check F | TGCTCATTGCGCCTGTGACAGCAAG |
| P*_cyd_-rhtA* check R | TTCGGATCGACGAGTTCATCCTGG |

Table S2 Abbreviation Index

| Abbreviation | Full Name |
| --- | --- |
| G6P | Glucose-6-phosphate |
| F6P | Fructose-6-phosphate |
| GAP | Glyceraldehyde-3-phosphate |
| PYR | Pyruvate |
| OAA | Oxaloacetate |
| CIT | Citrate |
| ICI | Isocitrate |
| α-KG | α-Ketoglutarate |
| SUCC | Succinyl-CoA |
| SUC | Succinate |
| FUM | Fumarate |
| MAL | Malate |
| Ru5P | Ribulose-5-phosphate |
| X5P | Xylulose-5-phosphate |
| R5P | Ribose - 5 - phosphate |
| S7P | Sedoheptulose-7-phosphate |
| E4P | Erythrose-4-phosphate |
| Glu | Glutamate |
| 5-ALA | 5-Aminolevulinic acid |
| PBG | Porphobilinogen |
| CPⅠ | Coproporphyrin Ⅰ |
| UPENⅢ | Uroporphyrinogen III |
| CPENⅢ | Coproporphyrinogen III |
| PPⅨ | Protoporphyrin IX |
| CPⅢ | Coproporphyrin Ⅲ |
| PRPP | Phosphoribosyl pyrophosphate |
|  | Continued Table S2 Abbreviation Index |
| Abbreviation | Full Name |
| IMP | Inosine monophosphate |
| AMP | Adenosine monophosphate |
| ATP | Adenosine triphosphate |
| GMP | Guanosine monophosphate |
| G6P | Glucose-6-phosphate |
| GTP | Guanosine triphosphate |
| Gln | Glutamine |
| OMP | Orotate monophosphate |
| OA | Orotic Acid |
| UMP | Uridine monophosphate |
| UTP | Uridine triphosphate |
| CTP | Cytidine triphosphate |
| dUMP | Deoxyuridine monophosphate |
| dTMP | Thymidine 5’-Monophosphate |
| PPP pathway | Pentose Phosphate Pathway |
| TCA cycle | Tricarboxylic Acid Cycle |
| NSB pathway | Nucleotide Sugar Biosynthesis pathway |


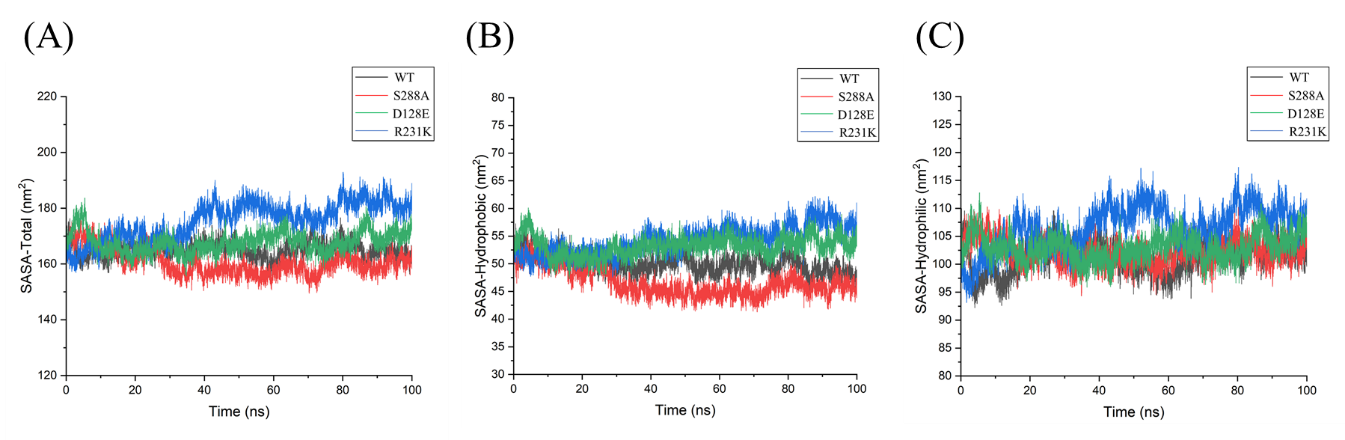


Fig. S1 Solvent Accessible Surface Area is used to describe the size of the solvent-contactable area on the surface of protein.


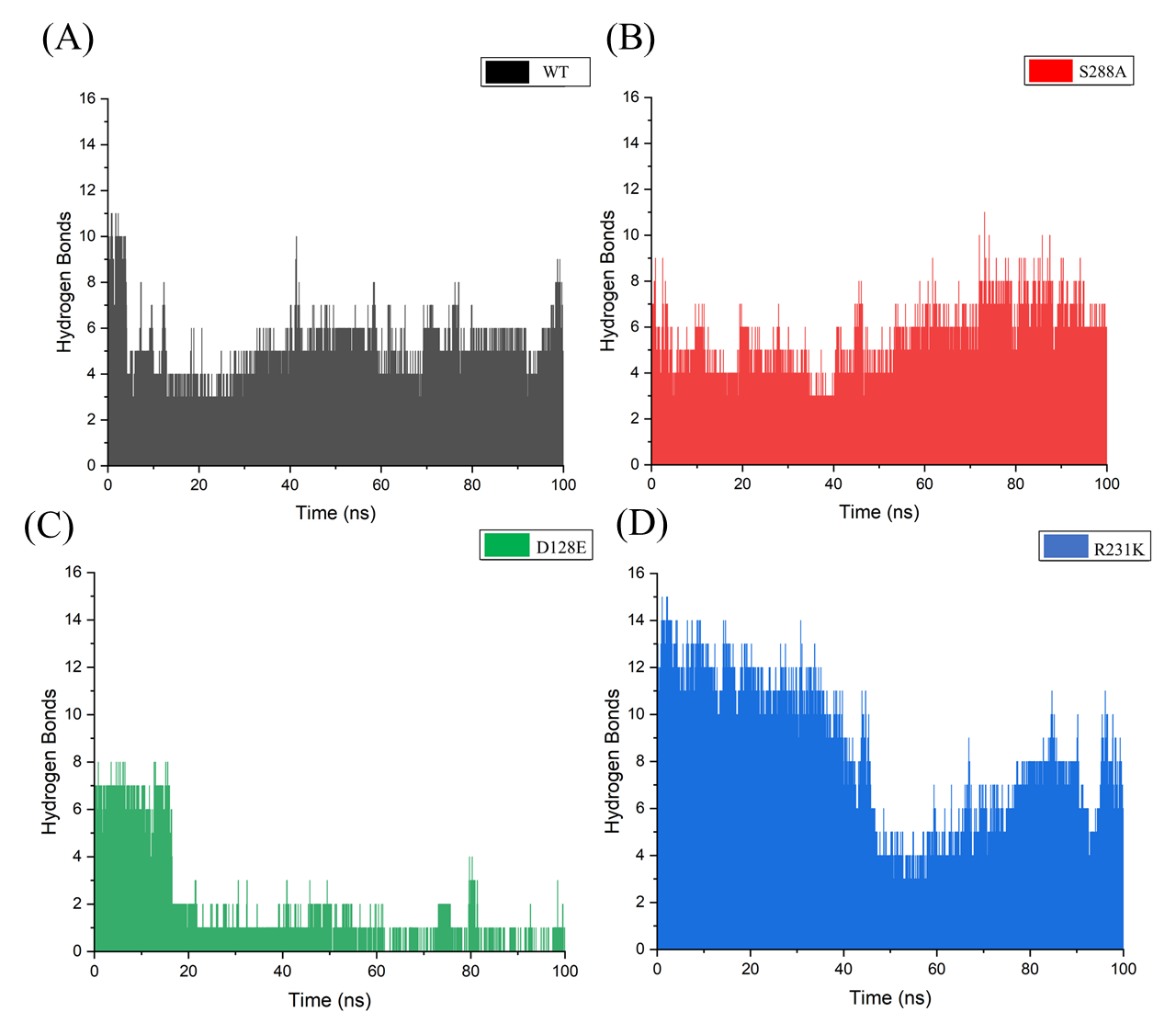


Fig. S2 Hydrogen Bonds is used to describe the formation and rupture of hydrogen bonds in protein simulation.
